# Comparing imaging, acoustics, and radar to monitor Leach’s storm-petrel colonies

**DOI:** 10.7717/peerj.6721

**Published:** 2019-04-30

**Authors:** Rachael A. Orben, Abram B. Fleishman, Abraham L. Borker, William Bridgeland, Amanda J. Gladics, Jessica Porquez, Peter Sanzenbacher, Shawn W. Stephensen, Roberta Swift, Matthew W. McKown, Robert M. Suryan

**Affiliations:** 1 Department of Fisheries and Wildlife, Hatfield Marine Science Center, Oregon State University, Newport, OR, United States of America; 2 Conservation Metrics, Inc., Santa Cruz, CA, United States of America; 3 Department of Ecology and Evolutionary Biology, University of California, Santa Cruz, CA, United States of America; 4 Oregon Coast National Wildlife Refuge Complex, U.S. Fish and Wildlife Service, Newport, OR, United States of America; 5 Oregon Sea Grant, Oregon State University—Extension Service, Astoria, OR, United States of America; 6 ABR, Inc.—Environmental Research & Services, Fairbanks, AK, United States of America; 7 Region 8—U.S. Fish and Wildlife Service, Palm Springs, CA, United States of America; 8 Region 1—Migratory Birds and Habitat Program, U.S. Fish and Wildlife Service, Portland, OR, United States of America; 9 Alaska Fisheries Science Center, Auk Bay Laboratories, Ted Steven’s Marine Research Institute, National Oceanographic Atmospheric Administration Fisheries, Juneau, AK, United States of America

**Keywords:** Aeroecology, Burrow-nesting, Deep-neural networks, Passive acoustic monitoring, Radar ornithology, Population monitoring, Machine learning, Seabird, Colonial breeder, Photo counts

## Abstract

Seabirds are integral components of marine ecosystems and, with many populations globally threatened, there is a critical need for effective and scalable seabird monitoring strategies. Many seabird species nest in burrows, which can make traditional monitoring methods costly, infeasible, or damaging to nesting habitats. Traditional burrow occupancy surveys, where possible, can occur infrequently and therefore lead to an incomplete understanding of population trends. For example, in Oregon, during the last three decades there have been large changes in the abundance of Leach’s storm-petrels (*Hydrobates leucorhoa*), which included drastic declines at some colonies. Unfortunately, traditional monitoring failed to capture the timing and magnitude of change, limiting managers’ ability to determine causes of the decline and curtailing management options. New, easily repeatable methods of quantifying relative abundance are needed. For this study, we tested three methods of remote monitoring: passive acoustic monitoring, time-lapse cameras, and radar. Abundance indices derived from acoustics and imagery: call rates, acoustic energy, and counts were significantly related to traditional estimates of burrow occupancy of Leach’s storm-petrels. Due to sampling limitations, we were unable to compare radar to burrow occupancy. Image counts were significantly correlated with all other indices, including radar, while indices derived from acoustics and radar were not correlated. Acoustic data likely reflect different aspects of the population and hold the potential for the further development of indices to disentangle phenology, attendance of breeding birds, and reproductive success. We found that image counts are comparable with standard methods (e.g., radar) in producing annual abundance indices. We recommend that managers consider a sampling scheme that incorporates both acoustics and imaging, but for sites inaccessible to humans, radar remains the sole option. Implementation of acoustic and camera based monitoring programs will provide much needed information for a vulnerable group of seabirds.

## Introduction

Globally seabird populations are declining; however this conclusion is largely driven by data on well monitored populations, the majority of which are surface nesting seabird species (Paleczny et al., 2015). Surface nesting seabirds are relatively easy to monitor at their breeding colonies because active nests can be identified and counted (e.g.,  Gaston, 2003). In contrast, much less is known about population trends of burrow nesting seabirds. Although species with this nesting behavior make up ∼50% of threatened seabird species (Spatz et al., 2014), these seabirds are much more cryptic at breeding sites, making surveys difficult (e.g.,  Piatt, Roberts & Hatch, 1990; Renner et al., 2006). Even in large colonies, studies are restricted to locations where nest cavities can be repeatedly accessed or where mark-recapture studies are feasible (e.g.,  Sheffield et al., 2006). In some cases, the mere presence of humans can damage nesting habitat or assist predators in locating nests (Rodway, Montevecchi & Chardine, 1996; Blackmer, Ackerman & Nevitt, 2004; Pollard, 2008). Additionally, there are often safety, logistic, and financial limitations to accessing remote seabird colonies. Therefore, alternative monitoring methods are needed. Most seabirds have k-selected life-history strategies (i.e., long-lived, few offspring per breeding attempt), and will skip breeding when conditions are poor, which means population monitoring programs need to be sustainable over decades to be viable. Thus, monitoring methods should be easily repeatable, statistically rigorous, and low cost. Additionally, methods need to be sufficiently robust to detect short-term changes in order to inform managers of population fluctuations prior to substantial declines. This need is desperate, as limited evidence suggests that burrow nesting seabird populations are declining globally, in part due to threats accrued on land (e.g., introduced predators, habitat degradation) (Croxall et al., 2012; Spatz et al., 2014). A lack of monitoring of this suite of species inhibits conservation prioritization and adaptive management to address global seabird declines.

Traditionally, burrow occupancy during the breeding period is used to measure reproductive effort. Burrow occupancy is determined using a ‘grubbing’ technique: visual or tactile inspection of burrows (sometimes with a burrow scope) in a given area. Though widely considered a reliable method (Parker & Rexer-Huber, 2016), potential for error remains, especially when investigators visit sites infrequently (e.g., once per season or less). Surveys are conducted during targeted periods of the breeding cycle (e.g., chick hatch), however annual variation in the timing of breeding can compound errors; for instance, if breeding failures occur prior to surveys this leads to underestimates of occupancy. Most colonies are large, requiring sub-sampling of burrows within standardized quadrats along a transect; this can lead to error if habitat is heterogeneous and not adequately sampled. Burrows can be convoluted networks with multiple entrances or unreachable nest chambers, leading to a high proportion of burrows with unknown status. Finally, despite efforts to standardize methods between surveys, observer biases have the potential to influence results through subtle differences in interpretation of protocols, and site-specific knowledge (e.g.,  Anker-Nilssen & Rostad, 1993). In spite of these caveats, burrow occupancy remains the simplest and most widely used metric of reproductive effort against which to assess the efficacy of newer monitoring methods.

*In-situ* environmental sensors have transformed ecological studies at all scales from individuals to populations (Burger & Shaffer, 2008; Marques et al., 2012). For population abundance monitoring, the use of acoustic recorders and remote cameras is rapidly growing, particularly for cryptic species (Blumstein et al., 2011; Trolliet, 2014; Gasc et al., 2016). These instruments can be deployed in the field and left to sample for long durations without costly human assistance. The number of species-specific vocalizations or images detected per unit of time can be used as an index of activity within the reception range of the recorder. Likewise, mobile marine radar can be used to monitor the activity of flying animals (Cooper et al., 1991). Advances in image capture and analytical programs have improved data acquisition from radar units (e.g.,  Assali, Bez & Tremblay, 2017). Improvements in current recording equipment and development of additional sensors will undoubtedly continue. Acoustic recordings, automated cameras, and radar have great potential for monitoring seabirds, but their relative efficacy has not been previously fully evaluated.

The high temporal and spatial resolution data recorded by electronic monitoring equipment makes manual review to identify species detections impractical. The lack of easily implemented automated methods to process these datasets currently limits the ability of ecologists to capitalize on the amount of data stored in image, video, acoustic, or radar files (Weinstein, 2017). However, data processing methods are rapidly improving. Machine learning and other automated detection algorithms can be applied to image and acoustic data sets to extract meaningful metrics (Deng, Hinton & Kingsbury, 2013; Cires˛an & Meier, 2015; Krizhevsky, Sutskever & Hinton, 2017). For instance, Deep Neural Networks (DNNs) can be used to detect sounds from recordings with spectro-temporal properties that are similar to signals produced by target species. Currently, some level of manual review is necessary to label data patterns corresponding to detections that are then used by classification algorithms to count all such patterns in a dataset. As new tools emerge, there is great promise in reanalyzing archived images and recordings. This, in itself, is an improvement on monitoring programs reliant solely on human collected data, as no visual or audio archive exists and time-series from these efforts are often constrained by evolving data collection methods.

It is important to compare the results of monitoring methods as each method has its own strengths and limitations. To date, there are few examples that compare abundance metrics for seabirds. Using visual counts of birds to infer population abundance is not a new approach (Bednarz et al., 1990; Clarke et al., 2003), but is context dependent. At the colony, counts can be used to document attendance patterns (e.g.,  Harding et al., 2005; Huffeldt & Merkel, 2013), however, doing so for species that arrive and depart from their colonies at night adds an additional challenge. Trials with infra-red video recording equipment at a European storm-petrel (*Hydrobates pelagicus*) colony found that flying birds were poor predictors of occupancy estimates derived from play-backs, likely because not all flying birds were related to the sample plots (Perkins, Bingham & Bolton, 2017). Acoustic call rates have rarely been compared to visual counts at seabird colonies. Acoustic call rates of surface nesting Forster’s terns (*Sterna forsteri*) yielded significant correlations with colony counts, however colony sizes were small, ranging from 15-111 breeding pairs (Borker et al., 2014). Finally, radar surveys have been used to measure changes in relative abundance of seabird populations for decades (Day et al., 2003; Cooper, Raphael & Peery, 2006; Raine et al., 2017), yet validation or comparative studies exist for a limited number of species (Bertram, Cowen & Burger, 1999; Cragg, Burger & Piatt, 2016).

The Leach’s storm-petrel, *Hydrobates leucorhoa*, (LESP) is a small burrow nesting seabird that lives 20 years or more (Morse & Buchheister, 1977). It breeds in dense colonies, typically located on off-shore rocks or islands across the Northern Hemisphere, with small numbers breeding in the Southern Hemisphere (Huntington, Butler & Mauck, 1996). Leach’s storm-petrels are highly vocal and nocturnally active at their breeding colonies, making them an ideal candidate species for comparing acoustic, image, and radar monitoring methods (Watanuki, 1986; Buxton & Jones, 2012). Additionally, conservation needs are pressing. Mark-recapture studies and play-back surveys in the North Atlantic have highlighted declines (Newson & Mitchell, 2008), but little is known about populations in the North Pacific. Infrequent surveys along the Oregon coast (1978, 1988, 2008) indicate that populations on some islands have dramatically declined while others have increased (Kocourek et al., 2009). However, the magnitude of the changes in population estimates, spatial inconsistencies, under-sampling of available habitat, and variability in survey timing have led to uncertainty about population status along the Oregon coast (Kocourek et al., 2009). As a result, researchers and managers are left with insufficient data to assess the conservation status and develop management plans. Therefore, there is a pressing need for monitoring methods that can be applied more frequently at multiple breeding colonies where effective conservation actions can be taken.

Here we compared four data collection methods to monitor abundance of Leach’s storm-petrels at their breeding colonies: monitoring by manual nest inspections (grubbing), infra-red cameras, acoustic recorders, and radar. We compared rates of burrow occupancy, to plot level abundance metrics (detection rates) from images and acoustics. We did not make a similar comparison with radar data because the radar collected information at a different spatial scale (i.e., colony-wide versus plot level). Then we compared the remote methods to each other. These monitoring methods provided data on different aspects of the population and at different spatial and temporal scales. Thus, we expected the strongest correlations among metrics that represent breeding performance (e.g., burrow occupancy and acoustic ground calls) or among metrics that represent population size (image and radar counts of flying birds and acoustic aerial calls), and poor correlation between these two groups. Finally, we discuss recommendations for future monitoring efforts of burrow nesting seabirds.

## Methods

### Study area

We established study plots in Leach’s storm-petrel (LESP) colonies on two offshore islands within the Oregon Islands National Wildlife Refuge on the Southern Oregon coast: Goat Island (42°4′1″N, 124°19′18″W, Oregon Colony Catalog #270-123; Naughton et al. 2007), a large colony with a dense breeding population (2012 breeding population estimate: 184,530; S Stephensen, pers. comm., 2018) and Saddle Rock (42°15′1″N, 124°24′53″W, Oregon Colony Catalog #270-079), a once large colony that experienced dramatic declines (2012 breeding population estimate: 2,504). Between 1988 and 2008, the colony at Saddle Rock declined from ∼87,000 to very few birds, however breeding birds still remained (Pollard, 2008; Kocourek et al., 2009). Goat Island was selected since it was currently the largest colony on the Oregon Coast. Studies were conducted on Goat Island in 2014 and 2015. In 2015, we added Saddle Rock to establish study plots at a site with a lower density LESP population (Table 1).

**Table 1:** Sampling periods for the methods used to measure abundance of Leach’s storm-petrels, *Hydrobates leucorhoa*, at two colonies in Oregon, U.S.A.

	**2014**	**2015**	
	**Goat Island**	**Goat Island**	**Saddle Rock**
Camera Deployment	April 9–Aug 20	May 5–Oct 7	June 19–Aug 27
Camera Sub-sampling^a^	B: July 11–17, 20–22	A & B: July 9–18	N & S: July 16–24
Radar	no survey	May 14, 17, 18, 21, 22 July 11, 13, 14	May 13, 15, 16, 19, 20 July 12^b^, 15, 16, 18, 19
Grubbing	July 16	July 16	July 15
Acoustics	A: April 9-Sept 11^c^ B: April 8-Sept 8 C: April 8-Aug 24	A: July 16–Oct 6 B & C: May 5–Oct 6	N: June 19–Aug 27 S: June 19–Aug 14

**Notes.**

a Cameras deployed on plot GIC were Moultries and these data were discarded from the study due to inferior image quality. In 2014, cameras on GIA had IR flash malfunctions and therefore did not record usable data.

b The radar data was contaminated by unknown interference on the night of July 12th and therefore excluded from analysis.

c In 2014, the GIA Song Meter was deployed with an incorrect clock (+3 h), the time associated with the recordings was adjusted, but recordings stopped ∼3 h prior to sunrise.

### Plot design

**Figure 1:**
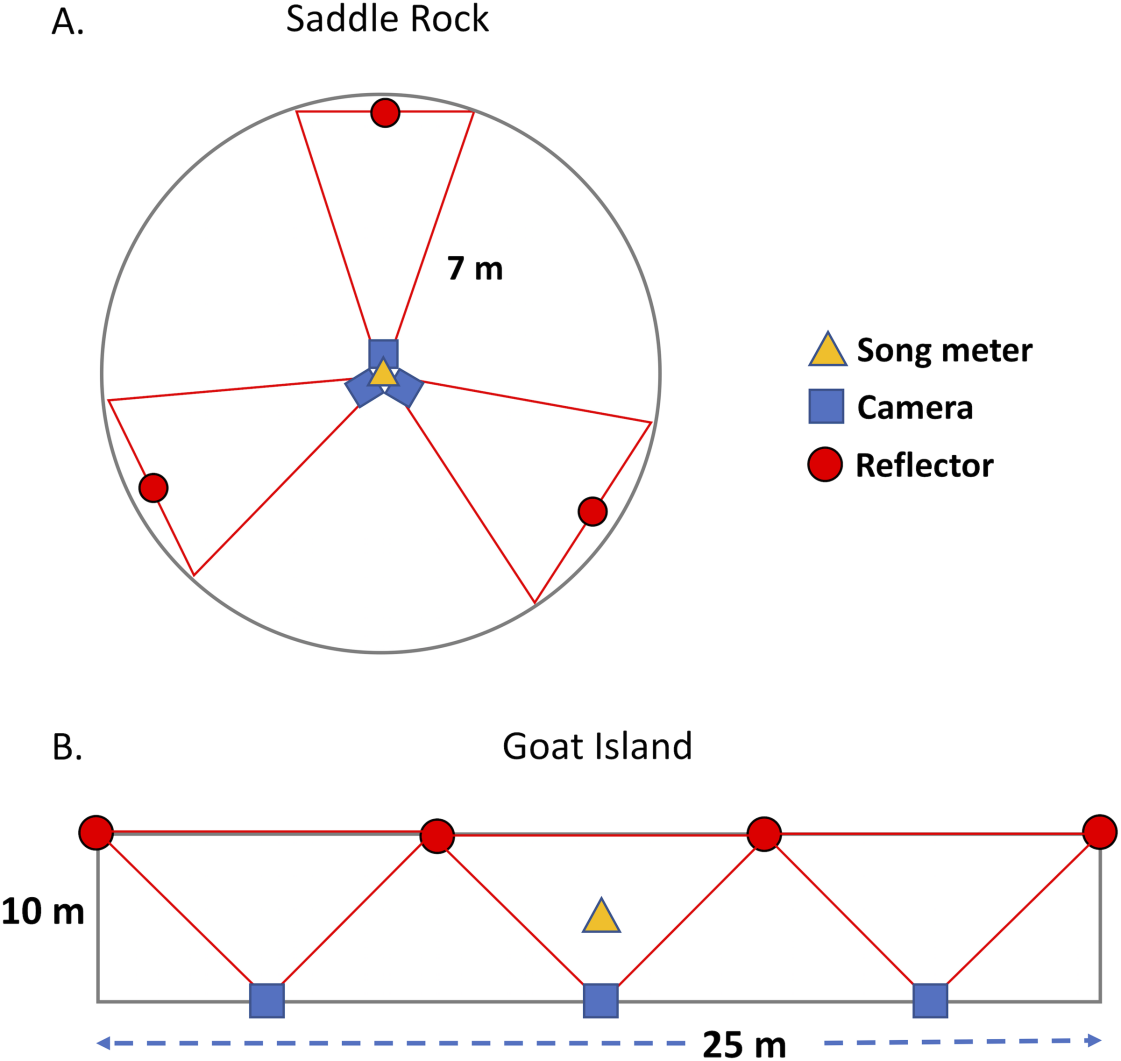
Plot design on Saddle Rock and Goat Island. A circular plot design was used on Saddle Rock; whereas, rectangular plots were used on Goat Island. The red lines indicate the approximate field of view for each camera.

On Goat Island, we deployed cameras and acoustic recorders and sampled burrow occupancy in three adjacent rectangular plots (10 m × 25 m) on the north vegetated slope (GIA, GIB, GIC, Fig. 1, Table 1). Each plot had three near-infrared cameras with self-contained covert illuminators (No-Glow) capable of taking single frame time-lapse or near video images (2 frames/sec). All cameras faced upslope to the south, away from the prevailing winds to minimize lens fouling; posts with reflectors marked the edge of the IR camera range (10.48 m) and are visible in the images. The cameras used were: Reconyx PC900 HyperFire (3.1 megapixels) and Moultrie M-990i (4 megapixels). Photos from the Moultrie cameras were of insufficient quality, potentially due to the IR flash, so were not used in the analysis. Cameras were powered by deep cycle 12V marine batteries, but recording was limited by 32GB SD cards. Cameras were attached 1.5 m above the ground to metal fence posts, padded with foam pipe insulation to minimize collision injury risk to birds. A single acoustic recorder (Song Meter SM3 with built-in microphones, Wildlife Acoustics, Concord, MA, U.S.A.) was positioned at the center of each plot at 0.3 m above the ground. Song Meters were powered with internal batteries or external battery packs depending on desired recording duration and contained 4 SD cards (128 GB each). At Saddle Rock, two circular plots (SRN and SRS) were set up with an acoustic recorder and three cameras mounted on a single post in the center (Fig. 1). Circular plots, with a radius of 7 m, were chosen at Saddle Rock because this generally matches the survey shape sampled by the acoustic recorders’ omnidirectional microphones. The area surveyed by an acoustic sensor is dependent on many factors including: ambient noise (wind, rain, surf), presence of target and non-target species, amplitude of target calls, relative humidity, and equipment and settings on the equipment. The sensor records a sphere. In a quiet location, that sphere might have a 100^+^ m radius, in contrast to a small 10 or 15 m radius in a loud location. The proximity of the plots (∼25 m) means they are not independent replicates in representing the entire island for comparison with radar, but they are mostly independent replicates for comparisons of cameras and acoustic recorders. Both islands were visited periodically throughout the summer to exchange SD cards and batteries and ensure equipment was operating correctly.

### Burrow surveys

Grubbing (manual inspection of nests) was conducted on both islands during early chick rearing following protocols from Kocourek et al. (2009) (Table 1). We randomly located three 1 m^2^ quadrats within each plot to determine burrow occupancy. Each quadrat was thoroughly searched by two individuals, and only burrow entrances with a clearly identifiable nesting chamber were included. All nest entrances within the quadrat were included even if the nest cavity was beyond the quadrat boundary. Due to low burrow densities, quadrat sampling at Saddle Rock yielded no burrows; therefore, entire plots were searched. No birds were removed from the nests, rather each nest was deemed ‘occupied’, ‘unoccupied’, or ‘unknown’; occupied nests was defined as having an adult, egg, or chick. Unknown nests were excluded from analyses. In 2015, we used a 1-inch diameter Pukamanu 1.0 burrow camera scope (Abyssal Hawaii, LLC) that allowed us to the assess occupancy status for all burrows during the second year of the study.

### Radar

In 2015, radar surveys were conducted from approximately sunset to sunrise for 3–5 nights during both late-May and mid-July at each colony associated with the new moon (Table 1). Surveys of Saddle Rock were conducted from Crook Point (42.250910°, −124.408100°), 500 m from Saddle Rock. The area surveyed included both Saddle Rock and North Crook Point Rock (Oregon Colony Catalog #270-076; Naughton et al. 2007). Surveys of Goat Island were also conducted from the mainland (42.072822°, −124.318605°), 600 m from the island. The radar laboratory consisted of two standard marine surveillance radar (Furuno Model FCR-1510; Furuno Electric Company, Nishinomiya, Japan) mounted on the roof of a truck (Cooper et al., 1991). The radar transmits at 9,410 MHz (i.e., X-band) through a 2 m long slotted wave guide, with a peak power output of 12 kW. We operated the radars concurrently with one oriented in horizontal mode (i.e., surveillance mode) and the other in the vertical mode. The surveillance radar antenna was tilted upward at ∼10° so that the bottom edge of the main radar beam was just below horizontal. Radars were operated at a range of 1.5 km with a pulse length at 0.07 µsec as these radar systems can detect Leach’s storm-petrels and similar-sized birds out to approximately 1.5 km (Sanzenbacher et al., 2010). We used automated image frame grabbers (model VGA2USB, Epiphan Systems Inc.) to record a high-quality, lossless image of the radar screen during each sweep of the radar (i.e., every 2.4 s).

### Instrument programming and data processing

#### Cameras

Reconyx cameras were programmed to take a 49.5 s burst of 99 frames every 10 min in 2014, and every 15 min in 2015, from 0100–0300 PDT. For comparisons with burrow occupancy we identified 10 clear nights closest to the grubbing date in each year (Table 1). We selected a subsample of 3 photo bursts within each night for density counts (GI 2014 *n* = 8910, 2015 *n* = 17,820; SR *n* = 13,365). Nights were only counted if >75% of photos in at least three bursts were clear enough to detect LESP. To assess the impact of frames lacking total clarity we classified LESP using four detection categories: (1) whether the bird was clear/easily recognizable, (2) only eye glare was present, (3) there was a distinguishable wing or body shape, or (4) only a blur was present. We counted detections per photo in each burst and then calculated mean bird detections per minute.

#### Acoustic sampling and processing

Acoustic recorders were programed to record continuously from local sunset to sunrise at a sample rate of 16,000 Hz (16 bit, stereo, .wav files). We developed automated classification models for two calls in the LESP vocal repertoire that are used in different behavioral contexts: the chatter and purr calls (Huntington, Butler & Mauck, 1996; Fig. S1). The chatter call is an accelerating bounding chatter producing a harmonic signal with a base frequency between 1,000 Hz and 2,000 Hz. The purr call is a repeated staccato buzz (800–1,000 Hz) ending with a convex ascending sweep between 750 Hz and 1,600 Hz. Males and females make both calls (Taoka et al., 1989). Birds in flight only vocalize with the chatter call, however birds on the ground or in burrows may also use this call; hereafter this call is termed the aerial call. The purr call is typically vocalized by birds in burrows and vocalization bouts can last more than 15 min (Taoka et al., 1988); hereafter this call is termed the ground call. Due to the differences in where birds make these vocalizations the aerial call likely propagates further, and has a greater detection range.

We automated the detection and quantification of aerial calls and ground calls on field recordings using custom software (implemented in Matlab 8.3). We split the field recordings into 2-second sound clips and measured the intensity of 10 spectro-temporal features in 187 separate frequency bins (43 Hz) within each 2-second clip at five different spectral resolutions. A spectral resolution is defined as a set of half-overlapping frequency bands. The five resolutions used in Hz were 312.5, 687.5, 1,437.5, 2,937.5, plus one band over the entire frequency range. These resulted in 10 feature types in each of 61 multiresolution frequency bands, for a full set of 610 candidate features. Each feature score is a combination of (1) spectral bin center, (2) spectral bin width (resolution), and (3) feature type (e.g., tone, transient, click).

DNN classification models were trained iteratively by adding examples of positive sounds (target calls) and representative non-target sounds from the soundscape at all survey sites. The DNNs learn patterns of spectro-temporal features that best differentiate target sounds from non-target sounds. The final model can then be applied to predict the probability that a target call is present in novel 2-second sound clips. Our training process uses a classification and regression tree (CART) algorithm to pre-select the best features for classification. The number of features chosen is a parameter that is automatically optimized in the training procedure. One limitation of this approach is that the presence of one LESP call in a 2-second clip is equivalent to a 2-second clip with multiple overlapping calls. Thus, in very busy soundscapes, this approach can become saturated when every 2-second clip contains a call, leading to situations where the addition of subsequent calls cannot be measured.

The performance of electret microphone elements in the SM3 internal microphones can be negatively impacted when saturated with water from repeated exposure to rain or water vapor. To detect potential microphone failures and remove poor quality recordings from the survey data, we created a metric of recording quality (named flux-sensitivity) that is a function of the average spectrum and the average fourth-power of the spectrum in each 2-second clip, as well as the 2 clips before and after each clip. The resulting spectral average falls somewhere between the average spectrum and the maximum spectrum in each clip. Flux-sensitivity ranges between 0 and 120^+^, and three situations will cause the value to be zero: (1) The microphone is dead and no signal is being registered, (2) The microphone is defective and is only registering a broadband electrical noise (fuzz), or (3) There is no bioacoustical activity present only wind noise, wave noise, or similar diffuse noise sources. To only remove data when the mic was not working correctly over a long period, all minutes with an average flux-sensitive value of 0 were removed from our analysis (851 min, 0.2%).

#### Aerial call classification model performance

Training data for the aerial call was generated by randomly sampling the recordings and manually reviewing and labeling 9,981, 2-second clips as containing LESP aerial calls or not (Table 2). Model selection was performed using cross-validation on a second labeled dataset (Table 2). Finally, a third and independent randomly sampled test dataset was used to measure model accuracy (i.e., ratio of positives to total detections) and sensitivity (Table 2). The accuracy of the aerial call model was also assessed through manual review of a random subset of 3% (∼140,755) of the 2-second clips in the entire dataset flagged as aerial calls by our model (accuracy: 98.7%). The aerial call model was then applied to the entire dataset (total = 6,806.41 h of recordings, or 12,226,008 2-sec clips). Classification model scores above the 99% probability threshold were accepted without manual review.

**Table 2:** Datasets used to for Deep Neural Network training, cross-validation and selection for detection of aerial calls and ground calls of Leach’s storm-petrels, *Hydrobates leucorhoa*. Each dataset consists of 2-second clips that contain either a positive or negative detection.

Call type	Positive detections	Negative detections	Accuracy^a^ (%)	Sensitivity^b^ (%)	Probability threshold^c^ (%)
Training dataset					
Aerial	6,004	3,977	–	–	–
Ground	6,149	20,883	–	–	–
Model selection dataset					
Aerial	4,002	4,051	99.7	85.33	99
Ground	4,095	15,617	85.96	52.62	50
Randomly sampled test dataset					
Aerial	1,357	3,414	98.35	78.92	99
Ground	151	4,630	75	23.84	50

**Notes.**

a Accuracy is calculated as the number of positive detections/(number of positive detections + number of negative detections) above a probability threshold.

b Sensitivity is calculated as the number of positive detections (above a probability threshold)/number of true positive events in the dataset. The number of true positive events was determined by manual review.

c The probability threshold is the user selected cutoff above which events are assumed to have the signal of interest.

#### Ground call classification model performance

Training data for the ground call model included 27,032-labeled 2-second clips (Table 2). Model selection was performed using cross-validation on a second independent dataset (Table 2). Because of low signal abundance in the data, we searched recordings from each site for additional examples of the ground call for inclusion in the model training and selection datasets, inflating the ratio of positive to negative examples (Table 2).

The randomly sampled test dataset only had 151 ground calls and the estimate of model sensitivity likely was not representative of model performance on the full dataset. In an attempt to correct for low signal abundance, 30-second clips centered on randomly selected ground calls from each site/year were compiled (*n* = 602; SRN and SRS plots only had 0 and 2 positive events respectively). Since a ground call presence is likely temporally correlated the 30-second window was chosen to increase the number of previously undetected ground calls in the dataset to help train the model. This resulted in 3,946 positives and 5,056 negative events; the final ground call model achieved 54.33% sensitivity at a 50% probability threshold. The ground call model was then applied to the entire dataset and all detections (111,389 events >50% probability) were manually reviewed to remove false positives. Real world accuracy on the entire dataset was 82.77% (92,197 positive detections).

#### Band limited energy analysis

Given the limitations of the call-based method in saturated soundscapes, we explored a separate independent metric of acoustic activity—band-limited energy analysis. Similar to a band limited energy detector (Mellinger et al., 2007), this approach can characterize calling intensity and is sensitive to overlapping calls, making it a useful approach when the signal of interest is the dominant signal in the soundscape, and overlapping calls prevent enumeration of additional individual calls above the level of saturation. The main limitation of this method is that band-limited energy analysis does not correct for the presence of non-target sound energy in the same frequency band as the target sound (e.g., broad-band noise from surf, wind, other species). In our case, and based on the previously reviewed acoustic clips, we assumed that the majority of the energy in this frequency range comes from LESP. To characterize the relative amount of acoustic energy in the frequency band where LESP aerial calls have the greatest amount of sound energy (1,376–1,462 Hz) we used a fast Fourier transform (window size = 372 overlap = 0.875) to measure mean relative energy in 187 separate 43-Hz frequency bins from 0–8,041 Hz for each 2-second sound clip. We then calculated the mean relative energy per bin per hour and took a mean of the bins from 1,376–1,462 Hz.

#### Radar analysis

At Goat Island, marine radar set in the horizontal (surveillance) position acquired a large amount of interference from ocean waves, therefore Goat Island survey results are from the vertical radar. Survey results from Saddle Rock are from the horizontal radar because this allowed for consistency with previous radar data from this site (Sanzenbacher et al. 2010). The sampling areas differ slightly between the two radar orientations but in the absence of interference radar counts were correlated. Across the five sampling days both radars collected data at Saddle Rock, *R*^2^ values ranged from 0.60–0.84 on four nights and was 0.24 on a single night, with the lower value likely influenced by weather and wave conditions. The abundance of storm-petrels in the airspace around colonies, combined with erratic flight patterns of these birds, made it difficult to discern individual flight trajectories. Therefore we used a pixel-based analysis as a measure of storm-petrel activity and abundance versus counting single radar targets (Bertram, Cowen & Burger, 1999). Specific methods and code for processing of radar images can be found in Sanzenbacher et al. (2010) and Sanzenbacher et al. (2017). Briefly, for each colony, a mask image was constructed to omit all pixels representing land and waves. Subsequently, LESP pixels were identified using a set of criteria describing the intensity of the radar signal (saturation 0-1, default 0.0; value 0-1, default 0.4) and size of contiguous pixels (diameter = 2). For each radar image the pixel count was summarized across 1-hectare grids at two scales surrounding each colony at 500 m and 50 m at 1-minute, and hourly periods. For the purposes of method comparisons only the large-scale (500 m) results are used as the two scales were highly correlated (nightly: *F*_1,15_ = 512.1, *R*^2^ = 0.97, *p* < 0.001; hourly: *F*_1,152_ = 5511, *R*^2^ = 0.97, *p* < 0.001). Construction of mask images and all radar data processing was automated using custom built scripts.

#### Method comparisons

For each method, we tested annual and island level differences. For annual differences in camera counts, we limited the dataset to when the moon was below the horizon. Then we used a linear-mixed model of the camera derived birds/photo/min in plot GIB, with year and moon phase as predictors and nested random effects (camera/night) (Pinheiro et al., 2018). To test for island differences, we used a similar model restricted to 2015 when both islands were sampled when the moon was above the horizon, with island and % moon illuminated as predictors and nested random effects (plot/camera/night). The timeframe of comparison during the moon cycle was different for the annual and island comparisons because comparative camera counts were available on different days (Table 1). For the acoustic data, we used a binomial GAM implemented in ‘mgcv’ to test for annual differences by accounting for the influence of diel, lunar, and seasonal patterns on hourly aerial call rates (0–30/hour) at Goat Island from May 7 to August 28 (Table 2) (Wood, 2011). We included night of year, hour of night, moon illumination, moon above and below the horizon, flux sensitivity, and the presence or absence of ground calls as predictor variables. We used a similar model for ground calls, but because ground call rates at Goat Island were low (mean = 0.54 calls/hour), we used the presence or absence of ground calls as the response variable. In this model we included aerial call rate as a predictor variable. We included the other type of call as a response variable in each model because one type of call could be obscuring the call of interest, or generally facilitating more vocal activity (Buxton et al., 2013).

Call rates (mean calls/min), acoustic energy, and camera detections (birds/min) were regressed against burrow occupancy to assess activity patterns relative to active nests for each year, island, and plot combination. To compare call rates with burrow occupancy, we calculated ground call rates for the majority of the night (440 to 120 min before sunrise), and aerial calls for two non-peak time periods of the night to try to avoid periods of saturated call rates: 120 to 240 min after sunset and 120 to 60 min before sunrise. Acoustic energy was calculated for each night. Mean call rate and acoustic energy were then calculated for a 29-night period centered on the date of grubbing; this allowed coverage for one complete lunar cycle.

We next assessed the strength of the correlations among radar, camera and acoustic measures of bird activity using simple linear regressions. Data were summarized as nightly rates and these were considered independent sampling events. The full extent of each overlapping timeframe between instruments was used for each method comparison. Comparisons among acoustic measurements (aerial call rates, ground call rates, acoustic energy), radar detections (large pixel scale), and camera detections (individual birds) were tested for the entire dataset at both colonies and for Goat Island alone to test for correlation at the high-density site only.

Due to the apparent saturation of aerial calls during periods of peak call activity, we tested the performance of a non-linear model to characterize the relationships between aerial call rates and nightly acoustic energy (Pinheiro et al., 2018), following the formula:

\begin{eqnarray*}\text{aerial call rate} \sim \frac{\text{asymptote}}{1+ \frac{\exp \nolimits (\text{xmidpt}-\text{response variable})}{\text{scale}} } \end{eqnarray*}

in which, xmidpt is equal to the half the saturation (15 call/min), asymptote indicates the value at saturation (30 call/min), and scale is the amount of change in the response variable needed to increase the aerial call rate from half to 3/4 (Bunnefeld et al., 2011). All analyses were conducted in R 3.3.2 (R Core Team, 2016), except where noted. Significance was set at *p* < 0.05.

## Results

### Burrow density and occupancy surveys

At Goat Island, total and occupied burrow densities were, respectively 5.44 burrows/m^2^ (±0.73 SE) and 4.6 burrows/m^2^ (±0.44 SE) in 2014 and 4.66 burrows/m^2^ (±0.57 SE) and 3.22 burrows/m^2^ (±0.29 SE) in 2015. In 2015, the burrow scope allowed for positive determination of 17 burrows that were deemed unknown using the grubbing method. Burrow occupancy was not significantly different between grubbing only and surveys augmented with a scope in 2015 (paired *t*-test, *t* = 0.15, *p* = 0.890, *df* = 3). At Saddle Rock, randomly placed quadrats yielded no burrows (*n* = 10 quadrats/plot). However, a census of both plots yielded a burrow density of 0.045 burrows/m^2^ (*n* = 14 burrows) with burrow camera assessed occupancy of 0.035 burrows/m^2^ (*n* = 11 occupied). These values were used in method comparisons. In 2014, the majority of occupied burrows on Goat Island contained small downy chicks without adults (75%). In 2015, 58% of occupied burrows at Goat Island contained downy chicks, some with adults. Similarly, at Saddle Rock 53% of occupied burrows contained downy chicks; whereas, the rest of the burrows had eggs or adults with undetermined nest contents.

### Camera surveys

In total, 24,966 storm-petrel detections from Goat Island and 126 from Saddle Rock were counted from camera images. Counting took 10–30 min for each burst containing 99 photos; counting time depended on bird density and image clarity. Of the detections on both islands, 74.7% were clear birds, 8.5% were eye shine, 6.0% were wings, and 10.9% were blurry (Fig. S2). However, detections on Saddle Rock were dominated by non-clear detection types. Using square root transformations for normality, detections of eye shine, wings, and blurry birds in each burst were positively correlated to clear bird detections (eye shine *R*^2^ = 0.34, *p* < 0.001; wings *R*^2^ = 0.21, *p* < 0.001; blurry birds *R*^2^ = 0.38, *p* < 0.001), but the low *R*^2^ values indicated loss of information when only clear birds were counted, therefore all detection types were combined. We elected to use counts from the entire 49.5 s (n_photos_ = 99) burst in our analysis. However, photos within each burst, were highly correlated with each other and subsampling of 11–50 photos/min led to R^2^ > 0.9, indicating that images could be collected at a lower frequency (Fig. S3). Other species were identified in the photos including western gulls (*Larus occidentalis*), night herons (*Nycticorax*), and rhinoceros auklets (*Cerorhica monocerata*). At Goat Island, camera detections were higher in 2015 (*F*_1,22_ = 18.62, *p* < 0.001). Camera detections were greater at Goat Island than Saddle Rock (*F*_1,2_ = 23.20, *p* = 0.041).

### Acoustic monitoring

From the full dataset, the aerial call model identified 4,691,985 events above the 99% probability threshold. In total, 92,200 ground call events were detected after manual removal of false positives. Because each 2 s clip was determined to contain a detection, or not, a maximum of 30 detections could occur in one minute. Of the minutes that contained aerial calls, 56.1% had >20 detections, which indicated frequent signal saturation for aerial call rates. For the ground calls, only 0.08% of minutes with calls had >20 detections.

**Figure 2:**
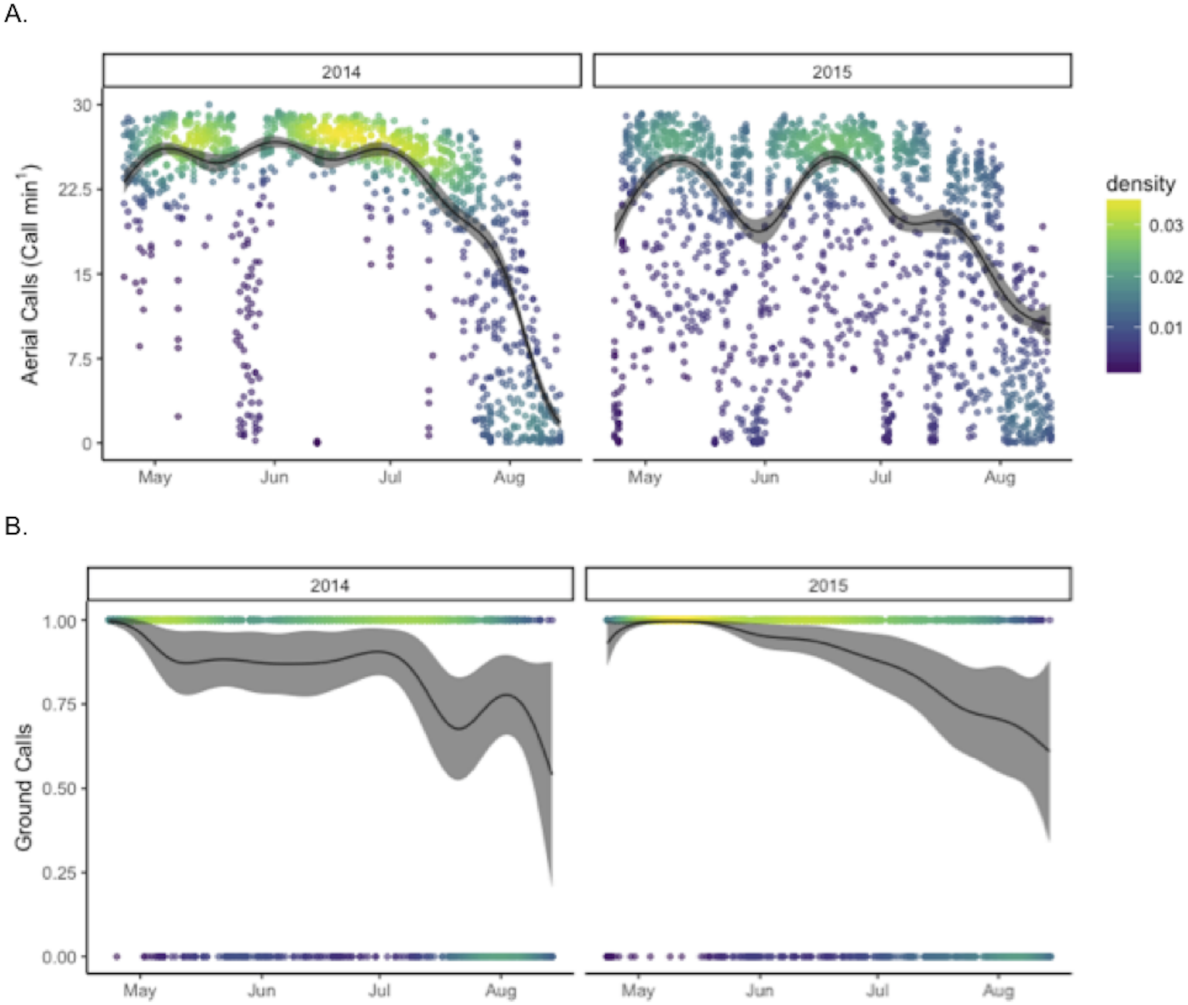
Annual patterns in call rates of Leach’s storm-petrels, *Hydrobates leucorhoa*, at Goat Island, Oregon. GAM estimates are shown for (A) hourly aerial call rates and (B) the presence (1) or absence (0) of ground calls in each hour. Model estimates were predicted by holding other contributing variables in the model constant at their mean value.

The final model explaining aerial call rates included night of year (by year), hour (22-4), moon illumination separated by whether the moon was above the horizon or not, presence of ground calls, and median flux sensitivity ( Table S1 and Fig. S5). This model explained 76.1% of the variation in aerial calls (Fig. 2), but retained a complicated structure of autocorrelation of fluctuating 2–3 day cycles (identified by visual inspection of autocovariance plots). Aerial call rates were significantly higher in 2014 than in 2015 (*p* < 0.001); similarly, hourly aerial call rates were relatively saturated (≥20 calls/min) more frequently in 2014 (61%), than in 2015 (23%) (*X*^2^ = 871.43, *p* < 0.001). The final binomial GAM model including night of year (by year), hour, moon illumination separated by whether the moon was above the horizon or not, aerial call rate, and median flux sensitivity explained 41% of the variation in ground call rates. At Goat Island, ground calls occurred more often in 2015 than in 2014 (*p* < 0.001). Ground call rates during hours when ground calls occurred were higher in 2015 (0.61 calls/min), than in 2014 (0.43 calls/min).

Binomial GAM models were run to test for differences in call rates between Goat Island and Saddle Rock in 2015 from May 5 to August 14 (Table 2). Both aerial call rates and ground call rates were significantly higher at Goat Island than at Saddle Rock (*p* < 0.001). The final model for presence of ground calls explained 71% of the variation and included island, night of year, hour (20-6), moon illumination separated by if the moon was above the horizon or not, aerial call rate, and median flux sensitivity (Table S1 and Fig. S5). The final model of aerial call rate explained 87% of the variation with same predictor variables except ground call presence replaced aerial call rate as a predictor variable.

### Radar Surveys

In total, over 209,000 radar images (average 11,000 images/night) were analyzed for LESP activity and summarized in 10-minute intervals relative to sunset. The radar detected storm-petrels during all 154 h of survey time. Nightly, the mean number of pixels/ha was low, and frequently zero, during the first hour after sunset and slowly increased with counts remaining high until the sixth or seventh hour after sunset and then decreasing until they were low or zero by sunrise. Summaries of monthly activity levels found that detections at Goat Island in May were 32.7 (±8.5 SE) pixels/ha and 33.7 (± 12.2 SE) pixels/ha in July. Detections at Saddle Rock were 1.04 (± 0.54 SE) mean pixels/ha in May and 2.39 (±1.03 SE) mean pixels/ha in July. Detections were significantly higher at Goat Island than Saddle Rock (*p* < 0.001, non-parametric two-sample Wilcoxon (Mann–Whitney)), whereas, detections were not different between May and July at either Goat Island (*p* = 1) or Saddle Rock (*p* = 0.063).

### Method comparisons

**Figure 3:**
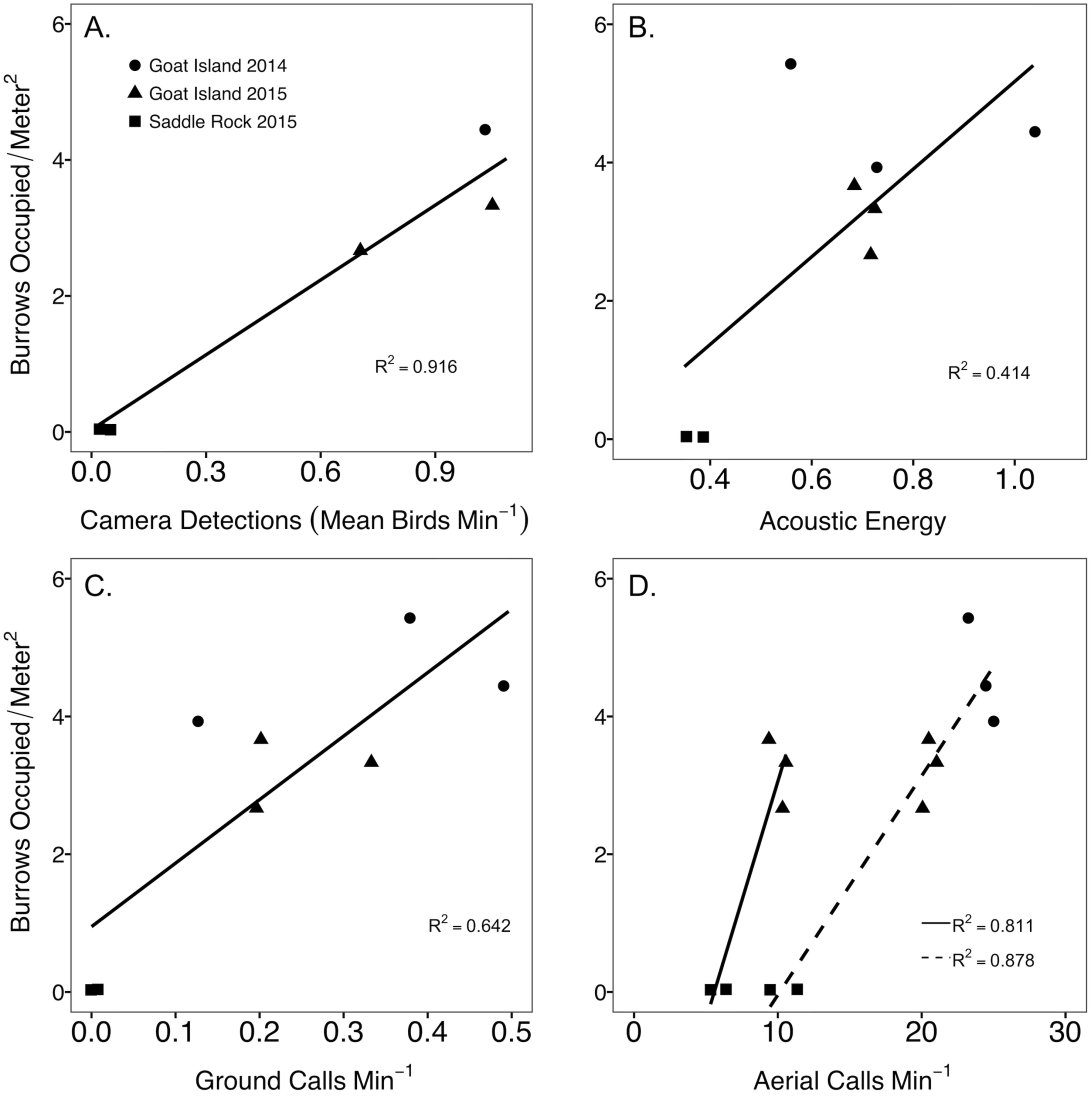
Comparison between abundance metrics of Leach’s storm-petrels, *Hydrobates leucorhoa.* Comparison between burrow occupancy and (A) camera counts, (B) acoustic energy, (C) nightly ground call detection rate, and (D) nightly aerial call detection rate for the period after sunset (120–240 min past sunset, dashed line) and a period prior to sunrise (60–120 min prior to sunrise, solid line) to avoid hours with high call saturation. Acoustic data was summarized for a 14-night buffer around the grubbing date to encompass one lunar cycle (29 nights). Ground call detection rates were calculated from 440 to 120 min prior to sunrise to encompass most of the night. In 2014, burrow occupancy was determined by manual inspection of the burrows, while in 2015 manual inspection was augmented with a burrow scope.

Annual estimates of camera detections and call rates of both aerial and ground calls were significantly related to burrow occupancy for each plot (Fig. 3). Camera detections were the most strongly correlated with burrow occupancy (*F*_1,3_ = 44.78, *p* = 0.007, *R*^2^ = 0.916), followed by aerial calls 120–240 min past sunset (*F*_1,6_ = 51.5, *p* = 0.0004, *R*^2^ = 0.878), aerial calls 60 to 120 min prior to sunrise (*F*_1,3_ = 18.21, *p* = 0.024, *R*^2^ = 0.811), and ground call rates (*F*_1,6_ = 13.56, *p* = 0.010, *R*^2^ = 0.642). Acoustic energy was not significantly related to burrow occupancy (*F*_1,6_ = 5.936, *p* = 0.051, *R*^2^ = 0.414). Because aerial calls during the post sunset time period neared call saturation, with >20 calls/min in 2014 (Fig. 3C), the aerial call rates prior to sunrise were used for further method comparisons. Island and year sample size was too limited to compare radar abundances with burrow occupancy, however higher radar detection rates mirrored higher burrow occupancy at Goat Island.

**Figure 4:**
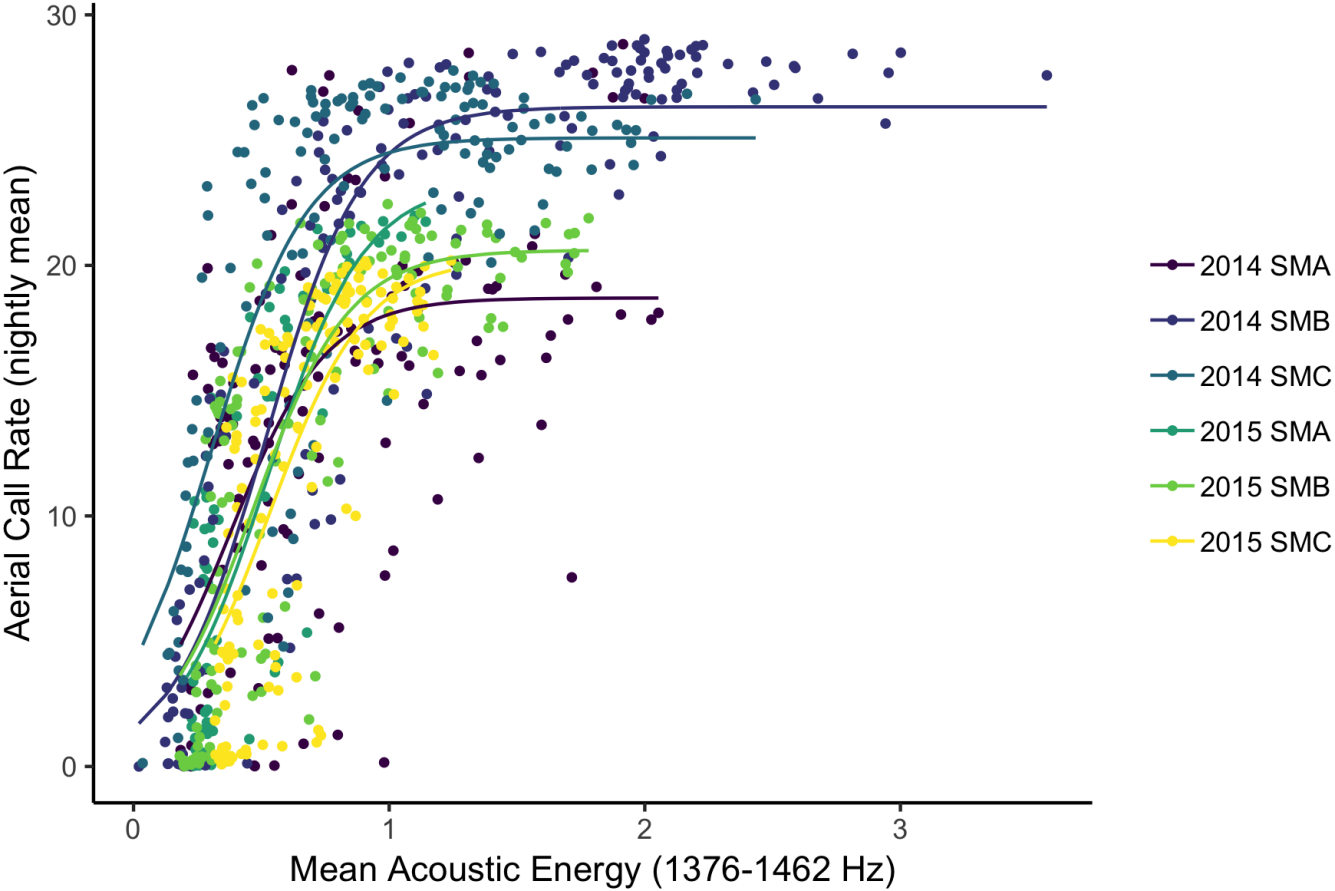
Acoustic energy at a Leach’s storm-petrel colony. Non-linear model fits between nightly acoustic energy from 1,376–1,462 Hz and nightly aerial call rates for the three plots on Goat Island, OR in both 2015 and 2016.

Using data from both islands, radar detection, camera detections, acoustics (call rates and energy) were significantly and positively correlated with the exception that radar detections did not correlate with either aerial or ground call rates (Table 3). Camera detections were significant and linearly correlated to all other metrics of abundance (Table 3). Acoustic energy was significantly related to both camera and radar detections; however, these correlations had relatively low *R*^2^ values. Aerial calls and camera detections were the most strongly related abundance metrics, and this was the only significant relationship between data collection methods using the high-density Goat Island data alone (Table 3). Generally, ground and aerial call rates were not highly correlated (Table 3, Fig. S4). Finally, to assess if acoustic energy was capturing additional information when acoustic call rates were saturated we fit non-linear models to the relationship of nightly aerial call rates with acoustic energy at Goat Island. Non-linear models improved AIC scores and provided a better fit to the data than linear models (Fig. 4).

**Table 3:** Summary of instrument comparisons for measuring nightly abundance of Leachs storm-petrels, *Hydrobates leucorhoa*, on Goat Island and Saddle Rock, Oregon. Linear models were run with both islands combined and then on Goat Island separately to test method comparisons at a high-density site only. Model estimates are sorted by *R*^2^ values; significant *p*-values are in bold. Radar models were restricted to 2015, while other models include data from both years.

**Comparison**	*R*^2^	*R*^2^**(adj.)**	**F**	***p***	**df**
**Both Islands**					
Aerial calls ∼ Camera	0.649	0.639	66.58	**<0.001**	36
Radar ∼ Camera	0.572	0.529	13.35	**0.004**	10
Aerial calls ∼ Acoustic Energy	0.512	0.511	991	**<0.001**	946
Ground calls ∼ Camera	0.312	0.297	20.86	**<0.001**	46
Camera ∼ Acoustic Energy	0.270	0.255	17.05	**0.0006**	46
Radar ∼ Acoustic Energy	0.207	0.175	6.526	**0.0171**	25
Aerial calls ∼ Radar	0.166	0.114	3.18	0.093	16
Ground calls ∼ Acoustic Energy	0.106	0.105	112.6	**<0.001**	946
Ground calls ∼ Aerial calls	0.102	0.101	107.8	**<0.001**	946
Ground calls ∼ Radar	0.048	−0.012	0.801	0.384	16
**Goat Island only**					
Aerial calls ∼ Acoustic Energy	0.559	0.558	1048	**<0.001**	827
Aerial calls ∼ Camera	0.457	0.427	15.14	**0.0011**	18
Radar ∼ Camera	0.188	−0.015	0.925	0.391	4
Camera ∼ Acoustic Energy	0.125	0.093	3.983	0.0558	28
Aerial calls ∼ Radar	0.119	0.039	1.48	0.249	11
Ground calls ∼ Aerial calls	0.092	0.091	83.94	**<0.001**	827
Ground calls ∼ Radar	0.094	0.012	1.141	0.308	11
Ground calls ∼ Acoustic Energy	0.084	0.082	75.44	**<0.001**	827
Ground calls ∼ Camera	0.075	0.042	2.258	0.1441	28
Radar ∼ Acoustic Energy	<0.001	−0.059	0.002	0.968	17

## Discussion

The metrics derived from acoustics, radar, and images each provided unique information on the abundance patterns of Leach’s storm-petrels, as none of the metric comparisons resulted in an *R*^2^ greater than 0.65. Counts of birds within the small field of view of the IR trail cameras were significantly correlated with all other metrics. Acoustic recorders were easily implemented throughout the season, and yielded three metrics of abundance: ground call rates, aerial call rates, and acoustic energy. Acoustic energy and aerial call rates were strongly related, but neither related strongly to ground calls. While nightly attendance patterns were similar between call rates and radar, overall correlation was low, potentially due to the scale of inference from each method or bird behavior (e.g., frequency of calls vs. flight). Long-term deployments of all methods have the potential to be the foundation of a robust monitoring program, if they can be implemented at the spatial and temporal scale necessary to document the population of interest. Further investigation is warranted to better understand the scale of change, or the sensitivity of each method to changes in abundance.

Trail cameras provided a promising new method for quantifying colony abundance of nocturnal seabirds. Counts from photos were strongly correlated with both the colony scale radar abundance index and the plot level burrow occupancy. Adding cameras to a monitoring program is low cost and requires minimal additional effort. With maturing automated image analysis, this tool will only improve. Likewise, implementing IR cameras as a stand-alone option seems viable as a low budget option, however there is significant species variation in flight patterns around colonies and which would likely influence the efficacy of this metric for other species (e.g., Perkins, Bingham & Bolton, 2017). Leach’s storm-petrels are small, agile fliers and appear to repeatedly take flight during the night when attending the colony. In contrast, heavier wing-loaded species such as sooty shearwaters (*Ardenna grisea*), or tufted puffins (*Fratercula cirrhata*) circle a colony, potentially relative to wind directions, and then land, exhibiting little localized flight behavior. These species-level differences in flight behavior are presumably also relevant considerations for both radar and acoustic metrics. An added benefit of IR cameras is that this method allows for detection of unexpected species and behaviors. In our case, we identified additional species including potential avian predators. This could also be done with acoustic recordings by running a suite of detectors for other vocally active species.

Overall, our findings suggest that among the current technology tested, acoustic monitoring has the greatest potential to monitor abundance of LESP at fine temporal resolution with multiple indices over long survey periods. While time-lapse photography and radar both could be scaled to deliver seasonal data, they provide a metric of aerial activity, albeit on different spatial scales per sensor (with one caveat, occasionally birds in images were counted on the ground). The information-dense acoustic recordings could yield a greater variety of indices beyond what we calculated. For instance, Leach’s storm-petrels exhibit sexual dimorphism in the frequency of their chatter calls that could provide insights into sex attendance patterns (Taoka et al., 1989), and detection of chick calls, heard during spot checks of the recordings, could provide an index of breeding success and a reference for breeding phenology. Additionally, unaccounted for short-term daily correlation remained in the binomial GAM analysis of acoustic call rates (e.g., 2–3 day cycles). This pattern may be related to foraging trip durations or social facilitation of colony attendance and further investigation is warranted (e.g., Granadeiro et al., 2009).

In this study, marine radar provided data at the colony scale, yet was most correlated to counts from cameras with a small spatial area of inference, potentially because both are metrics of flight activity. This, and the lack of correlation between radar and ground calls suggests that flight behavior measured at the colony scale may not be tightly linked with reproductive effort. This is perhaps not surprising as individual flight-paths may be convoluted and at the colony-scale includes non-breeding birds. However, further investigation is needed to disentangle this. Additionally, at the colony scale, breeding seabirds continue to attend even when reproductive success is low and non-breeding seabirds may visit colonies throughout the breeding cycle, often with a seasonal peak in the return of failed breeders and prospecting immatures (Boulinier et al., 1996). Little specific information is available for colony attendance of non-breeding birds for Leach’s storm-petrels and this complicates interpretation of any abundance metric tested in our study. As implemented, radar created the least disturbance, since colonies were not physically visited and remains the sole option for inaccessible sites. With the advances in technology that have occurred since we conducted our study the maintenance visits for cameras and acoustic recorders would not be necessary; regardless, colonies need to be accessible for deployment and retrieval of recording devices. Given the varying results of the method comparisons, coupled with the costs of purchasing and maintaining radars and the greater complexity of radar operations relative to camera and acoustic units, we do not recommend additional radar studies for LESP along the Oregon coast at this time.

A reliable long-term monitoring method must be robust to shifts in breeding phenology. For burrow nesting seabirds, surveys are traditionally timed to coincide with assumed periods of high colony attendance (Ratcliffe et al., 2010), yet seabird breeding phenology is known to shift due to changing environmental conditions (Gjerdrum et al., 2003; Frederiksen et al., 2004). This consideration is certainly pertinent for the Oregon coast, as recent oceanographic conditions—a marine heatwave (Bond et al., 2015), are unprecedented (Frölicher & Laufkötter, 2018). Very little is known about how these changes in marine conditions could impact storm-petrel breeding phenology in Oregon, but seabird breeding phenology is often variable (Byrd et al., 2008; Youngflesh et al., 2018). Using a monitoring method that captures the full extent of the breeding season, prevents missed information and misinterpretation. As implemented here, both camera and acoustic monitoring provide the temporal data stream necessary to address this, but further analysis is needed. Stationary radar systems could be set-up to record a similar seasonal record, but this effort is much more logistically complex than deploying acoustic recorders and trail-cameras.

Understanding annual differences in abundance is highly dependent on an understanding of within season variation in attendance patterns. Throughout the season, individual Leach’s storm-petrels visit the colony for different reasons and at different frequencies. For example, during incubation, when mate switches might occur infrequently, aerial calls, photo counts, and radar surveys might underestimate population size, but provide a better understanding of the number of breeders. Conversely, during a colony re-occupation period a higher number of non-breeders might visit for nest prospecting and pair bonding and result in higher population estimates and greater variability among monitoring metrics. For instance, the cross-method comparison revealed low *R*^2^ values between ground calls and all other metrics, yet correlation with burrow occupancy, suggesting this metric is related to breeding effort rather than colony attendance. Additionally, annual or colony level differences in foraging trip durations (Pollet et al., 2014; Hedd et al., 2018), could lead to differences in abundances of birds attending a colony on a given night. We found a relationship with moonlight for both ground and aerial call rates, this was not completely surprising as avoidance of colonies on full moon nights has been previously documented (Watanuki, 1986; Buxton et al., 2013). Our hourly analysis of acoustic and radar data highlight the night pattern in colony attendance by Leach’s storm-petrels: an increase, plateau, and drop-off by dawn. When testing for annual differences it is important to obtain a season-long record of attendance to avoid mismatches in phenology between years. Other explanatory factors such as weather conditions influence colony attendance patterns of seabirds and detection rates of acoustic recorders and cameras, and inclusion would likely increase the explanatory power of our models.

Analytical methods are rapidly evolving, and could be adapted to overcome two of the analytical challenges we found in this study: overlapping aerial calls and manual counting of photos. We found an annual differences in aerial call rates, however this difference was largely driven by the lower percentage of saturated hours in 2015. Saturation of aerial call rates was the result of how the acoustic data were processed, and in future studies this could be overcome by reducing the 2-second call detection window, applying a sliding window, and/or building detectors that are able to count overlapping calls. Acoustic energy appeared to overcome some of the challenges in aerial call saturation, but provides a less precise metric as ambient sounds, especially wave noise, also contributed to and complicated the record at Saddle Rock. Similar to advances in acoustic signal recognition, methods to automate the recognition and subsequent counting of objects in photos are rapidly evolving. During our study, camera counts were limited in scope due to the extensive amount of time that manual counting required. Ideally, detection algorithms for automated counting of target images that are currently under development will substantially reduce this processing time (Weinstein, 2017).

## Recommendations

The success of a long-term monitoring hinges on a robust sampling design and the availability of reliable resources to sustain the monitoring effort. We did not include a comprehensive analysis of the cost of each of the methods since they are continually changing and survey effort impacts costs, but equipment costs (cameras < acoustic recorders < radar), field costs (cameras ≈ acoustic recorders < radar), data processing (radar < automated acoustic call rates < manual camera counts), and data interpretation (radar ≈ camera counts < acoustic data) all contribute to method feasibility and how easily a given monitoring method could be implemented on a scale representative of seabird populations.

Whereas acoustic recorders are currently more commonly used in remote monitoring of seabird colonies, we recommend a new emphasis on deployment of cameras for a number of reasons. First, camera derived counts were correlated with acoustic metrics, radar surveys and burrow occupancy estimates (more data is needed to better understand these relationships). Thus, camera derived counts could be particularly helpful for monitoring less vocal species such as tufted puffins (*Fratercula cirrhata*). Second, photos have the ability to detect rare events or species (e.g., predators) that could be informative for management in unanticipated ways (Anderson et al., 2017). The same is true for acoustic datasets since the sampling sphere is larger; however, particular sounds need to be filtered through detection algorithms or spectrograms need to be visually reviewed. In contrast, photos may be more easily scanned for the unexpected. We found camera counts were highly correlated over the duration of a burst. It seems likely that photographs taken at 1–10 min intervals throughout the night would yield as much or more information on abundances than the targeted counts of this study. A more evenly spaced recording scheme throughout nighttime hours would allow trail cameras to record for a longer duration. Finally, since we purchased trail cameras, improvements on image capacity and battery life make this application more feasible without maintenance visits, and increased demand may foster purpose-built camera systems.

However, we do not imply validation and interpretation of detection rates from acoustics and images are complete. Additional validation studies should be done concurrently with monitoring over the longer-term because datasets can be re-analyzed as methods progress. Our method comparisons appeared to be related at large effect sizes (i.e., when data from both colonies was included). Likewise, our sample sizes were small and additional calibration at intermediate densities is needed, along with a plot design that better reflects instrument sampling range. Our analysis identified significant differences between years. These differences may be reflective of variation in attendance patterns related to colony success. Our analysis was limited to 2–3 paired camera and acoustic recorders per-colony, per-year. This appears adequate to capture large-scale differences in abundance, but spatial placement and the number of recorders relative to the size of the colony are key considerations. The number of recorders that are needed per unit area still needs validation and is beyond the scope of this study, but likely is dependent on species characteristics and colony topography (e.g., Oppel et al., 2014). Placement of recorders at colony edges versus colony centers could lead to different conclusions depending on how population changes occur (colony expansion/contraction versus more uniform changes in density). Additionally, placement of acoustic recorders must consider other sources of ambient noise that could interfere with recording quality such as wind and waves (Buxton & Jones, 2012). Likewise, placement of cameras needs to consider prevailing winds and weather to maximize collection of usable photos. In our study, we coupled the two to make direct comparisons, but deploying instruments in an alternating grid could capture more spatial variation.

Our results indicate that IR camera and acoustic monitoring have potential for low cost monitoring of dense colonies of burrow or crevice nesting species. Additionally, results of our acoustic monitoring suggest that with additional analysis and advances in technology acoustics could provide an efficient monitoring tool in these scenarios. Considering the lack of information on population trends and the need to identify declines before they are catastrophic, we urge managers to employ both methods while working towards refining detection and data analysis methods. Indeed, the USFWS Inventory and Monitoring Programs of the US West Coast regions are currently developing seabird monitoring protocols under the new Pacific Seabird Program and are considering these methods as they reach the full implementation stage for each species. Remote methods analyzed in this study have the potential to provide low impact, repeatable, and low cost monitoring techniques for annual monitoring and, thereby, provide an early warning indicator and the opportunity to discover and mitigate the root causes of population changes.

##  Supplemental Information

10.7717/peerj.6721/supp-1 Figure S1 Supplemental figures and tables Supplemental figures and tables for the manuscript: Comparing imaging, acoustics, and radar to monitor Leach’s storm-petrel colonies

10.7717/peerj.6721/supp-2 Data S1 Burrow occupancy of Leach’s storm-petrels on Goat Island and Saddle Rock, Oregon Burrow occupancy was determined via grubbing and in 2015, manual inspection was supplemented by a burrow scope. Occupancy is indicated by occupied (O), unoccupied (U), unknown (X) or not checked (NA). Contents were indicated as bird (B), bird and downy chick (BCs), bird and egg (BE), dead chick (Cd), feathered large chick (Cl), downy chick (Cs), egg (E), dead egg (Ed), not checked (NA), unknown (U), or confirmed empty (X). Entrance characteristics were coded as multiple entrances (ME) or single entrance (SE). Passage structure was coded as multiple passages (MP), single passage (SP) or unknown (U).

10.7717/peerj.6721/supp-3 Data S2 IR camera images from Leach’s storm-petrel colonies in Oregon, USA Photos were taken with Reconyx PC900 HyperFire trail cameras every 10 minutes in 2014, and every 15 minutes in 2015, from 0100 –0300 PDT. Each sampling event lasted for a 49.5 sec burst recording 99 frames.

10.7717/peerj.6721/supp-4 Data S3 Radar survey target densities from Leach’s storm-petrel colonies in Oregon, USA Radar surveys were conducted from approximately sunset to sunrise for 3-5 nights during both late-May and mid-July at both Saddle Rock (Crook Point) and Goat Island using standard marine surveillance radar (Furuno Model FCR-1510; Furuno Electric Company, Nishinomiya, Japan).

10.7717/peerj.6721/supp-5 Data S4 Ground call rates of Leach’s storm-petrels from Goat Island and Saddle Rock colonies in Oregon, USA Acoustic recorders (Song Meter SM3 with built-in microphones, Wildlife Acoustics, Concord, MA, U.S.A.), were used to collect the raw acoustic records. Then each record was segmented into 2-second clips and Deep Neural Networks (DNNs) were used to identify storm-petrel ground calls (purr call).

10.7717/peerj.6721/supp-6 Data S5 Aerial call rates of Leach’s storm-petrels from Goat Island and Saddle Rock colonies in Oregon, USA Acoustic recorders (Song Meter SM3 with built-in microphones, Wildlife Acoustics, Concord, MA, U.S.A.), were used to collect the raw acoustic records. Then each record was segmented into 2-second clips and Deep Neural Networks (DNNs) were used to identify storm-petrel aerial calls (chatter call).

10.7717/peerj.6721/supp-7 Data S6 Acoustic energy from Leach’s storm petrel colonies in Oregon, USA To characterize the relative amount of acoustic energy from 1376-1462 Hz, we used a fast Fourier transform (window size = 372 overlap = 0.875) to measure mean relative energy in 187 separate 43-Hz frequency bins from 0-8041 Hz for each 2-second sound clip. We then calculated the mean relative energy per bin per hour and took a mean of the bins from 1376-1462 Hz.

## Additional Information and Declarations

### Data Availability

The following information was supplied regarding data availability: The processed data is available for radar, acoustic call rates, acoustic energy, camera counts, and burrow surveys in the Supplemental Files.
