# Supplementary material for: Comparing imaging, acoustics, and radar to monitor Leach’s storm-petrel colonies
Source: PeerJ. 2019 Apr 30;7:e6721. doi: 10.7717/peerj.6721 (PMC6499055; doi:10.7717/peerj.6721)
Supplement: Figure S1 — Supplemental figures and tables for the manuscript: Comparing imaging, acoustics, and radar to monitor Leach’s storm-petrel colonies [file peerj-07-6721-s001.docx]

**
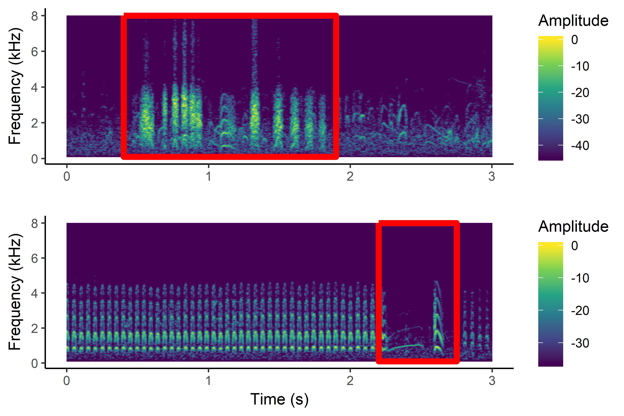
**

Supplemental Figure 1. Leach’s storm-petrel calls: A) aerial chatter call, B) purr ground call spectrograms with the targeted spectral signal in the red box on both panels.


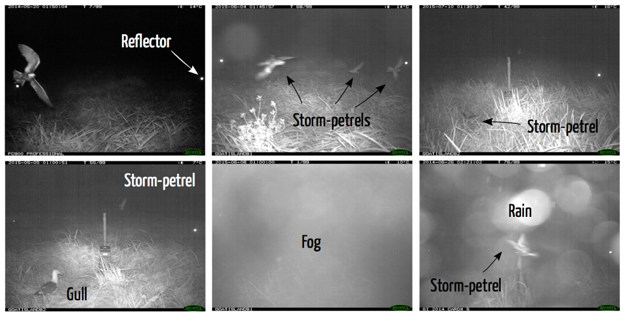


Supplemental Figure 2. Examples of photos from the Reconyx trail cameras. The quality of infrared photographs varied widely, with some clearly showing storm-petrels in the air and on the ground. Other images were entirely obscured by fog or rain.


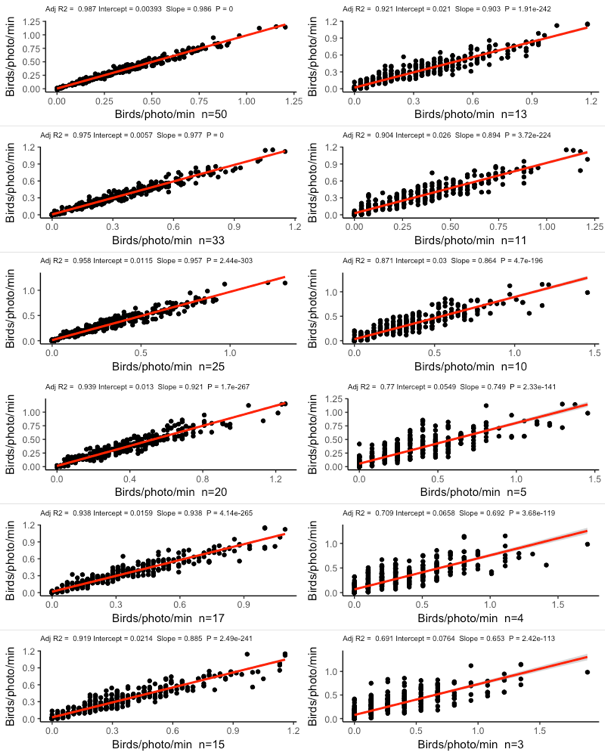


Supplemental Figure 3. Correlation in storm petrel detections within each camera burst (99-photos). On each plot the sample size in the x-axis (n) is the number of photos used for a comparative estimate. The y-axis represents the entire burst.


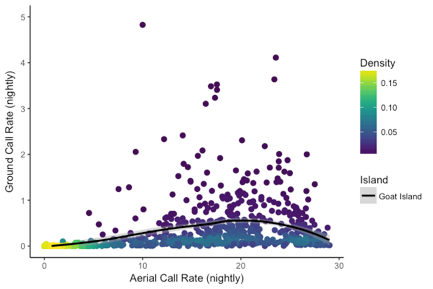


Supplemental Figure 4. Non-linear relationship between nightly aerial and ground call rates at Goat Island. on Oregon Coast National Wildlife Refuge during summer 2014 and 2015. Due to low ground call rates at Saddle Rock, there is not an apparent pattern. A loess smoother was applied to the data to illustrate the non-linear relationship.

Supplemental Table 1. Results of final generalized additive models for assessing temporal patterns of call rates of Leach’s storm petrels, *Hydrobates leucorhoa,* between years at Goat Island and between Goat Island and Saddle Rock in 2015. Edf: estimated degrees of freedom for each smoother, Res.df: residual degrees of freedom, % Dev: percent deviance explained, R^2^ = adjusted pseudo-R^2^. Plots were initially included in all models, but the term was removed during AIC model selection. Hours are shown in local time, but were adjusted to be a continuous variable for the models (e.g. 1-10).

|  | **Edf** | **Res.df** | **Chi.sq** | ***p*** | **% Dev** | **R^2^ (adj.)** |
| --- | --- | --- | --- | --- | --- | --- |
| **Aerial Call Hourly Rates (year)** |  |  |  |  | **76.8** | **0.797** |
| Night of year (2014) | 8.996 | 8.999 | 4540.5 | <0.001 |  |  |
| Night of year (2015) | 8.754 | 8.953 | 2371.9 | <0.001 |  |  |
| Hour (22-4) | 5.970 | 6.000 | 4267.2 | <0.001 |  |  |
| Moon Illumination, moon down | 7.534 | 8.188 | 71.12 | <0.001 |  |  |
| Moon Illumination, moon up | 7.989 | 8.606 | 242.3 | <0.001 |  |  |
| Flux sensitivity median | 8.886 | 8.996 | 3733.9 | <0.001 |  |  |
| Year | - | - | 334.7 | <0.001 |  |  |
| Moon up | - | - | 101.7 | <0.001 |  |  |
| Ground calls (Y/N) | - | - | 294.1 | <0.001 |  |  |
|  |  |  |  |  |  |  |
| **Aerial Call Hourly Rates (island)** |  |  |  |  | **87.3** | **0.9** |
| Night of year | 8.886 | 8.989 | 1765.7 | <0.001 |  |  |
| Hour (20-6) | 5.936 | 5.998 | 6036.3 | <0.001 |  |  |
| Moon Illumination, moon down | 8.618 | 8.921 | 153.7 | <0.001 |  |  |
| Moon Illumination, moon up | 7.808 | 8.552 | 169.0 | <0.001 |  |  |
| Flux sensitivity median | 8.496 | 8.905 | 5519.9 | <0.001 |  |  |
| Ground calls (Y/N) | - | - | 250.6 | <0.001 |  |  |
| Island | - | - | 1296.1 | <0.001 |  |  |
|  |  |  |  |  |  |  |
| **Presence of Ground Calls (year)** |  |  |  |  | **41** | **0.449** |
| Night of year (2014) | 7.769 | 8.429 | 44.379 | <0.001 |  |  |
| Night of year (2015) | 7.568 | 8.268 | 156.954 | <0.001 |  |  |
| Hour (22-4) | 4.372 | 5.163 | 76.071 | <0.001 |  |  |
| Moon Illumination, moon down | 3.41 | 4.211 | 4.723 | 0.3828 |  |  |
| Moon Illumination, moon up | 5.353 | 6.42 | 17.183 | 0.0097 |  |  |
| Flux sensitivity median | 5.148 | 6.332 | 52.418 | <0.001 |  |  |
| Aerial call rate | 7.094 | 8.147 | 179.211 | <0.001 |  |  |
| Year | - | - | 23.93 | <0.001 |  |  |
|  |  |  |  |  |  |  |
| **Presence of Ground Calls (island)** |  |  |  |  | **71** | **0.744** |
| Night of year | 6.886 | 7.697 | 81.850 | <0.001 |  |  |
| Hour (20-6) | 4.931 | 5.553 | 48.998 | <0.001 |  |  |
| Moon Illumination, moon down | 1 | 1 | 0.152 | 0.697 |  |  |
| Moon Illumination, moon up | 7.940 | 8.673 | 27.508 | 0.0003 |  |  |
| Flux sensitivity median | 3.068 | 3.905 | 20.874 | 0.0003 |  |  |
| Aerial call rate | 3.031 | 3.78 | 63.610 | <0.001 |  |  |
| Island | - | - | 65.8 | <0.001 |  |  |

A.

B.

Supplemental Figure 5. GAM estimates of annual, seasonal, and diel trends in call rates of Leach’s storm petrels, *Hydrobates leucorhoa*, at Goat Island, Oregon as shown for A) hourly aerial call rates and B) hourly ground call rates.
